# Amivantamab, a bispecific epidermal growth factor receptor and mesenchymal-epithelial transition factor inhibitor, associated with ulcerative intertrigo

**DOI:** 10.1016/j.jdcr.2025.08.035

**Published:** 2025-09-19

**Authors:** Hannah R. Concannon, Robyn G. Ku, Morgan Brazel, Maria Angelica Selim, Amber Fresco

**Affiliations:** a Duke School of Medicine, Durham, North Carolina; b Duke Department of Dermatology, Durham, North Carolina; c Duke Department of Pathology, Durham, North Carolina

**Keywords:** adverse drug reaction, amivantamab, drug eruptions, EGFR inhibitor, granuloma inframammary adultorum, intertrigo, MET inhibitor, tyrosine kinase inhibitors

## Introduction

Amivantamab is a bispecific antibody used in the treatment of nonsmall cell lung cancer targeting epidermal growth factor receptor (EGFR) and mesenchymal-epithelial transition factor (MET) that has known dermatologic adverse effects (dAEs). As EGFR is involved in regulating keratinocyte growth and differentiation, the most common dAEs of EGFR inhibitors include xerosis, pruritus, acneiform eruptions, and paronychia.^1^^,^^2^ MET inhibitors are associated with the same dAEs in addition to mucositis. Due to its dual mechanism of action, it is thought that amivantamab may cause more severe dAEs, with case reports of severe dAEs of the scalp, erosive lesions of the penis and inguinal and perianal regions, and symmetrical drug-related intertriginous and flexural exanthema.^1^^,^^3^, ^4^, ^5^, ^6^

## Case report

**Fig 1:**
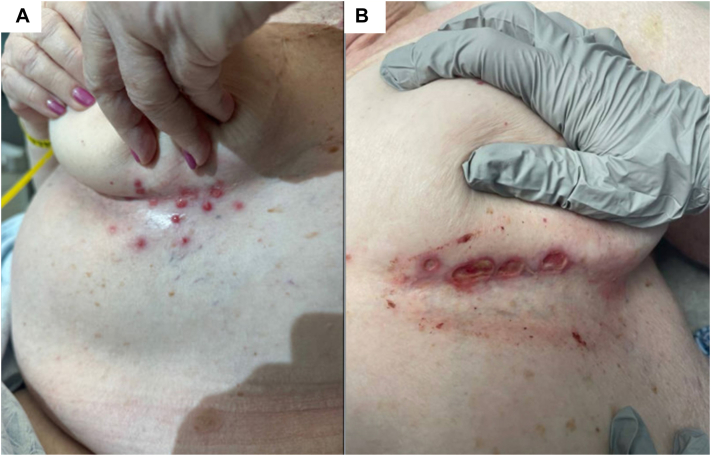
**A,** Eroded papules in the left inframammary fold at first visit with dermatology. **B,** Ulcerative intertrigo in the left inframammary fold at dermatology follow-up during second flare.

We report the case of a 75-year-old woman who was referred to Dermatology for a full body rash that developed after 3 months of treatment for stage IV lung adenocarcinoma with amivantamab 1400 mg infusions every 3 weeks. Prior to referral, she was prescribed oral fluconazole, topical nystatin, and hydrocortisone cream. At presentation, she had an acneiform eruption of the face, chest, and upper back as well as painful paronychia of the right great toenail. Additionally, there were eroded erythematous papules of her inframammary folds (Fig 1, *A*), groin, and buttocks. Culture swab of a groin lesion showed *Pseudomonas oryzihabitans*. Polymerase chain reaction tests for varicella zoster virus and herpes simplex 1 and 2 viruses were negative. Amivantamab treatment was paused due to dAEs, and she was prescribed a 2-week course of doxycycline 100 mg twice daily (for concomitant paronychia and acneiform eruption) and an empiric course of valacyclovir (until polymerase chain reaction results were negative) and instructed to perform dilute vinegar soaks of the affected intertriginous areas. After 2 weeks, skin examination showed dramatic improvement.

**Fig 2:**
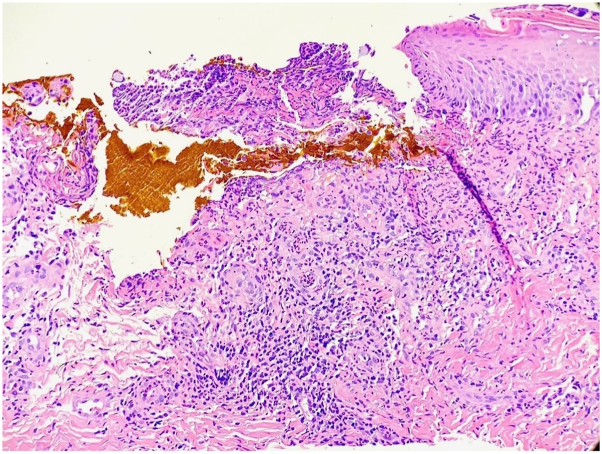
H&E-stained slide showing epidermal ulceration with associated mixed inflammation and dermal fibrosis (20×). *H&E*, Hematoxylin and eosin.

Three months later, the patient resumed a half-dose amivantamab treatment (700 mg every 3 weeks) due to tumor growth with a prophylactic course of doxycycline prior to infusion to reduce the risk of dAE. The patient returned to clinic 2 weeks after infusions resumed for a milder but similar acneiform eruption. She also reported return of rash in her axillae and groin, although intertrigo was not noted on examination. She discontinued her amivantamab treatment again after 3 months due to rash and arthralgias. Six weeks after her last infusion, the patient was seen in Dermatology clinic for recurrence of inframammary (Fig 1, *B*) and groin ulcers. On examination, she was found to have erythematous, punched-out ulcerations and coalescing papules in the inframammary folds and groin. Tissue culture of an inframammary ulcer was negative for bacteria, atypical mycobacteria, and fungus. Biopsy showed epidermal ulceration with associated mixed inflammatory infiltrate of neutrophils, lymphocytes, and eosinophils and granulation-type tissue in the superficial dermis with surrounding dermal fibrosis (Fig 2). Per patient report, the rash cleared within a week of treatment with hydrocortisone and has not reoccurred after starting quarter-dose amivantamab treatment (350 mg every 3 weeks).

## Discussion

Our patient developed recurrent erosive and ulcerative intertriginous eruptions reminiscent of granuloma inframammary adultorum and granuloma gluteale adultorum, forms of erosive papulonodular dermatosis thought to result from irritant dermatitis associated with skin occlusion, especially in patients requiring diapers or using excessive occlusive topicals.^7^^,^^8^ The name refers to macro-appearance; biopsy does not show granulomas. Instead, biopsy typically shows dense, mixed intradermal infiltrate with nonspecific histology.^8^ A similar eruption was described in a patient treated with amivantamab for nonsmall cell lung cancer who developed penile and inguinal ulcers.^6^ Unlike symmetrical drug-related intertriginous and flexural exanthema, a recognized dAE of amivantamab, granuloma inframammary, and gluteale adultorum present with discrete, erythematous to violaceous eroded papules and nodules rather than erythematous patches.^3^ Although the punched-out ulcerations could be consistent with pyoderma gangrenosum, they appeared in an atypical location for pyoderma gangrenosum and biopsy did not show characteristic neutrophilic infiltrate.

A common factor between this patient’s unusual rash and dAEs associated with amivantamab is the presence of granulation tissue on biopsy. One case series describes Erosive Pustular Dermatosis-like eruptions in patients taking amivantamab that showed “florid granulation tissue” on biopsy, which may be linked to dysfunctional growth and differentiation of keratinocytes caused by EGFR inhibition.^4^ Additionally, suggestive of a causal link between amivantamab and this patient’s ulcerative intertrigo is the appearance of a dose-dependent relationship, as she had a recurrence of a milder rash with half-dose amivantamab and no recurrence with a quarter dose.

This patient’s medical history includes diabetes mellitus, type II, and obesity, which both contribute to the development of intertriginous rashes.^7^ Notably, she previously experienced a rash beneath her breasts and in her groin after treatment with mobocertinib, another tyrosine-kinase inhibitor. At the time, it was presumed to be a yeast infection; mobocertinib treatment was stopped for other reasons and a Dermatology referral was never placed. The prior rash associated with mobocertinib may indicate patient-specific risk for dAE in intertriginous regions, which could be linked to known risk factors for intertriginous rash.

## Conclusion

This case of ulcerative intertrigo associated with amivantamab may present a new example of a unique side effect of this combination EGFR-MET inhibitor: ulcerative lesions, particularly in intertriginous areas. Awareness of this adverse effect may allow patients to continue potentially life-saving therapy and help clinicians choose therapies less likely to cause severe dAEs.

## Conflicts of interest

None disclosed.
